# Changes of the Proteome and Acetylome during Transition into the Stationary Phase in the Organohalide-Respiring *Dehalococcoides mccartyi* Strain CBDB1

**DOI:** 10.3390/microorganisms9020365

**Published:** 2021-02-12

**Authors:** Franziska Greiner-Haas, Martin von Bergen, Gary Sawers, Ute Lechner, Dominique Türkowsky

**Affiliations:** 1 Institute of Biology/Microbiology, Martin-Luther University Halle-Wittenberg, 06120 Halle (Saale), Germany; franzigreinerhaas@gmail.com (F.G.-H.); Gary.Sawers@mikrobiologie.uni-halle.de (G.S.); 2 Department of Molecular Systems Biology, Helmholtz Centre for Environmental Research–UFZ, 04318 Leipzig, Germany; martin.vonbergen@ufz.de

**Keywords:** *Dehalococcoides mccartyi*, growth phase, proteome, organohalide respiration, chlorobenzene, mass spectrometry, Nε-lysine acetylation, reductive dehalogenation, Tat transport, anaerobic respiration

## Abstract

The strictly anaerobic bactGIerium *Dehalococcoides mccartyi* obligatorily depends on organohalide respiration for energy conservation and growth. The bacterium also plays an important role in bioremediation. Since there is no guarantee of a continuous supply of halogenated substrates in its natural environment, the question arises of how *D. mccartyi* maintains the synthesis and activity of dehalogenating enzymes under these conditions. Acetylation is a means by which energy-restricted microorganisms can modulate and maintain protein levels and their functionality. Here, we analyzed the proteome and Nε-lysine acetylome of *D. mccartyi* strain CBDB1 during growth with 1,2,3-trichlorobenzene as an electron acceptor. The high abundance of the membrane-localized organohalide respiration complex, consisting of the reductive dehalogenases CbrA and CbdbA80, the uptake hydrogenase HupLS, and the organohalide respiration-associated molybdoenzyme OmeA, was shown throughout growth. In addition, the number of acetylated proteins increased from 5% to 11% during the transition from the exponential to the stationary phase. Acetylation of the key proteins of central acetate metabolism and of CbrA, CbdbA80, and TatA, a component of the twin-arginine translocation machinery, suggests that acetylation might contribute to maintenance of the organohalide-respiring capacity of the bacterium during the stationary phase, thus providing a means of ensuring membrane protein integrity and a proton gradient.

## 1. Introduction

*Dehalococcoides mccartyi* is a strictly anaerobic bacterium that relies exclusively on the reductive dehalogenation of organohalides for energy conservation and growth. Growth is also restricted to the use of acetate plus CO_2_ as carbon sources and hydrogen as the electron donor. Organohalide respiration (OHR) is based on an outwardly facing multi-protein complex located in the cytoplasmic membrane. It consists of the cobalamin (coenzyme B_12_)-containing catalytic subunit RdhA and the putative membrane anchor RdhB of the reductive dehalogenase as the terminal electron-accepting enzyme, the electron-delivering H_2_-uptake NiFe hydrogenase (HupSLX), and the subunits OmeA and B of an organohalide respiration-associated molybdoenzyme [1,2]. Since quinones are not involved in the electron transfer from hydrogen to the reductive dehalogenases [3], it has been hypothesized that electron transport through the dehalogenating protein complex generates a proton gradient, with the integral membrane protein OmeB being a possible site for proton translocation [4,5].

*D. mccartyi* strain CBDB1 [6] was shown to reductively dehalogenate an exceptionally broad spectrum of halogenated, mostly aromatic, electron acceptors ranging from polychlorinated to -brominated benzenes (e.g., [7,8], polychlorinated dibenzo-*p*-dioxins [9], biphenyls [10], phenols [11], including bromophenol blue [12], and the herbicide 2,4,5-T [13]). Despite its slow growth, with doubling times between 1 and 3 days [14], *D. mccartyi* is a key organism for the bioremediation of groundwater and soil contaminated with organohalides [15]. Its highly specialized energy metabolism is reflected by the large number of 32 non-identical reductive dehalogenase-homologous (*rdhAB*) genes in *D. mccartyi* strain CBDB1. So far, only one of its RdhAs, CbrA, has been functionally characterized as a chlorobenzene reductive dehalogenase, catalyzing the dechlorination of 1,2,3-trichlorobenzene (1,2,3-TCB) to 1,3-dichlorobenzene [16]. As proteomic studies have demonstrated, CbrA was highly expressed in hexachlorobenzene-dechlorinating cultures, along with another RdhA bearing the locus tag cbdbA80. A further 14 RdhA proteins were detected in the proteome, however, at lower abundances [17]. Transcription analyses revealed that in the presence of 1,2,3- or 1,2,4-TCB, all 32 *rdhA* genes were induced, albeit in amounts spanning several orders of magnitude. These studies also showed that *cbrA* was among the most highly expressed *rdhA* genes [18]. Although these data indicate a major role played by transcriptional control mechanisms in response to organohalides, it was shown that the mRNA levels of specific OHR-related genes do not necessarily correlate with the abundance of the respective proteins [19]. This suggests that post-transcriptional or post-translational regulation might influence the abundance and activity of these gene products in *D. mccartyi* and contribute to the adaptation of the strain to changing environmental conditions, such as a fluctuating supply of halogenated electron acceptors. This is of particular importance in view of the energy and resources required to synthesize the comparatively highly abundant and large OHR multiprotein complex and to transport it through the cytoplasmic membrane [1].

The acetylation of the ε-amino group of lysines is now recognized as a ubiquitous and dynamic mechanism for post-translational modification of many proteins in all domains of life, and acetylation/deacetylation is a mechanism whereby protein function can be modulated [20]. Since the first study of a bacterial acetylome [21], studies have expanded to cover several Gram-positive and Gram-negative bacteria. These studies have revealed an abundance of acetylations in the enzymes of central metabolic pathways, of transcription factors, and of structural proteins, but they also revealed variation of the acetylation profiles with changing growth conditions [22,23]. The degree of Nε-lysine acetylation results mainly from the interplay of acetyltransferases and deacetylases using acetyl-coenzyme A (acetyl-CoA) or acetylphosphate as substrates, but can also originate from a non-enzymatic reaction [22].

*D. mccartyi* strain CBDB1 is predicted to encode nine GNAT (Gcn5-related *N*-acetyltransferase) family acetyltransferases and one Zn-dependent deacetylase in its genome, suggesting that protein acetylation might play a role in the adaptation to changing environmental conditions. Recently, the acetylome of the facultative organohalide-respiring bacterium *Sulfurospirillum halorespirans* was analyzed [24]. Interestingly, the response regulator and histidine kinase of a two-component system involved in the regulation of *rdhA* gene regulation were shown to be acetylated. This acetylation was related to the observed long-term “retentive memory” of *rdhA* gene expression in the absence of a chlorinated compound. In the current study, we studied the proteome and acetylome of *D. mccartyi* strain CBDB1 in the exponential and stationary growth phases. Particular attention was paid to the coverage of functional pathways and to the detection of protein acetylation, which could identify key enzymes that are important in metabolic transition and survival in the stationary phase. The transcription of selected genes was also quantified throughout growth to elucidate the temporal role of transcriptional and post-translational regulatory processes potentially governing the observed strong synthesis and longevity of the OHR complex. Our findings suggest that acetylation conceivably plays a role in governing the maintenance of OHR-associated functions, thus aiding the long-term survival of *D. mccartyi*.

## 2. Materials and Methods

### 2.1. Growth Conditions and Experimental Design

*D*. *mccartyi* strain CBDB1 was cultivated anaerobically in Ti(III)-citrate-reduced (1.5 mM), carbonate-buffered synthetic medium at 30 °C under an atmosphere of nitrogen-carbon dioxide (80:20) with hydrogen as the electron donor (0.5 bar overpressure) and acetate (5 mM) as a carbon source [6]. The cultures (60-mL volumes) were incubated statically and overlaid with the electron acceptor 1,2,3-TCB dissolved in hexadecane (400 mM) (2.5% *v*/*v*, resulting in a nominal concentration of 10 mM). 1,2,3-TCB continuously partitioned from the overlaying hexadecane layer into the water phase (log10 partition coefficient between hexadecane and water: 4.04), resulting in a calculated initial aqueous concentration of ~36 µM [25]. Two proteomic experiments were conducted. In the first experiment, the proteomes and acetylomes were analyzed in the exponential and the stationary growth phases. In the second experiment, the proteomes were also analyzed in the exponential and stationary growth phase and additionally, in the inoculum (a 4-week-old stationary phase culture) and a freshly inoculated control culture incubated for five days without 1,2,3-TCB. Additionally, the transcription profiles of selected *rdhA* genes were monitored during growth. For each data point, three biological replicates were analyzed.

### 2.2. Nucleic Acid Extraction

From three replicates of the culture harvested in the stationary phase of experiment 2, samples were taken throughout growth for RNA (2 mL) and DNA (1 mL) extraction. The cultures were centrifuged (8415× *g* for 20 min at 4 °C); the cell pellets were resuspended in 100 µL of residual supernatant and stored at −80 °C until further processing. For DNA extraction, the NucleoSpin^®^ Tissue Kit (Macherey-Nagel, Düren, Germany) was used according to the instructions provided by the manufacturer. The DNA was eluted in 100 µL distilled H_2_O. A Total RNA Mini Kit (A&A BIOTECHNOLOGY, Gdansk, Poland) was used for RNA extraction. To correct for losses during RNA extraction and handling, 1 µL of Coleoptera Luciferase mRNA (9.9 × 10^8^ copies µL^−1^, Promega, Mannheim, Germany) was added to the cell pellet as an internal standard immediately before RNA extraction. The RNA was eluted with 100 µL distilled, diethylpyrocarbonate-treated H_2_O, treated with DNase I (NEB, New England Biolabs, Frankfurt (Main), Germany) to remove residual DNA and stored at −80 °C.

### 2.3. Reverse Transcription and Quantitative PCR (RT-qPCR).

cDNA was synthesized using the RevertAid^TM^ H Minus First Strand cDNA Synthesis Kit (Thermo Scientific, Schwerte, Germany) with random hexamer primers according to the manufacturer’s instructions. The synthesized cDNA served as a template for quantitative PCR amplification with primers (Table S1) targeting the genes of interest. The amplification of cDNA and DNA was performed in QuantiTect^®^ SYBR^®^ *Green* PCR master mixes (Qiagen, Hilden, Germany) and a primer concentration of 0.5 µM in the Rotor-Gene RG-3000 (Corbett Research, Sidney, Australia). Plasmids carrying fragments of the respective genes (Table S1) were serially diluted and included as external standards in the qPCR runs, as described previously [18].

### 2.4. Cell Harvest for Proteomic Analyses

A total cell count of at least 10^9^ for the proteome analyses, each as three biological replicates, was used. The cells were first centrifuged at 3500× *g* and at 4 °C for 45 min, then the supernatant was removed except for the last 2 mL. The cells were resuspended in the remaining supernatant, transferred to 2 mL Eppendorf tubes, and centrifuged again at 8415× *g* and at 4 °C for 30 min. The pellet was resuspended in 100 µL remaining supernatant and washed with 1.5 mL 1× phosphate-buffered saline (PBS) buffer (at 8415× *g* and 4 °C for 30 min). The cell pellets were stored in 100 µL residual supernatant and kept at −80 °C until further processing.

### 2.5. Peptide Preparation from Lysed Cells

Protein extraction, digestion, and peptide purification were performed as described before [26]. Briefly, cells were dissolved in 8 M urea lysis buffer (20 mM HEPES, 8M urea, 1 mM sodium vanadate, 1 mM β-glycerol phosphate, 2.5 mM sodium pyrophosphate) and disrupted by four cycles of freeze/thaw/ultrasonic bath treatment. Cell debris was removed by centrifugation (15 min, 4 °C, 20,000× *g*) and proteins were quantified by the BCA assay (Thermo Fisher Scientific, USA). After reduction (4.5 mM dithiothreitol, 30 min, 55 °C) and subsequent alkylation (10 mM iodoacetamide, 15 min, RT, in the dark), samples were diluted four-fold with 20 mM HEPES, pH 8. In experiment 1, three replicates each of 44 µg (exponential phase) and 117 µg (stationary phase) and in experiment 2, three replicates each of 60 µg (exponential and stationary phases, inoculum) and 3 µg (control culture) were digested overnight with an equivalent of 2% (*w*/*v*) trypsin (Promega, Mannheim, Germany). Digested peptides were acidified with 1% (*v*/*v*) trifluoroacetic acid (TFA), desalted over SEP PAK Classic C18 columns (Waters, Milford, MA, USA) and eluted using 40% (*v*/*v*) acetonitrile in 0.1% (*v*/*v*) TFA. For proteome analysis, 5% (*v*/*v*) of the eluate was used; all samples were lyophilized and stored at −80 °C.

### 2.6. Immunoaffinity Enrichment of Lysine-Acetylated Peptides

The PTMScan Acetyl-Lysine Motif Kit (Cell Signaling Technology, Beverly, MA, USA) was used to enrich acetylated peptides from the desalted peptides of experiment 1 according to the recommendation of the manufacturer. Briefly, peptides were dissolved in immunoaffinity purification (IAP) buffer and incubated with Kac-antibody beads for 2 h, at 4 °C. The beads were washed with IAP buffer and water, and acetylated peptides were eluted with 0.15% (*v*/*v*) TFA in two steps.

### 2.7. Liquid Chromatography-Tandem Mass Spectrometry

Non-modified peptides from experiments 1 and 2 and acetylated peptides from experiment 1 were desalted by using SOLAµ SPE plates (Thermo Fisher Scientific, Waltham, MA, USA). After solubilizing peptides in 0.1% (*v*/*v*) formic acid, they were separated using a 100 min non-linear gradient from 3.2% to 40% (*v*/*v*) acetonitrile and 0.1% (*v*/*v*) formic acid on a C18 analytical column (Acclaim PepMap100, 75 μm inner diameter, 25 cm, C18, Thermo Scientific, Waltham, MA, USA) in a UHPLC system (Ultimate 3000 RSLCnano, Dionex/Thermo Fisher Scientific, Idstein, Germany) at a flow-rate of 300 nL/min and 35 °C. Mass spectrometry was performed on an Orbitrap Fusion mass spectrometer (experiment 1) or a Q Exactive HF MS (experiment 2, both Thermo Fisher Scientific, Waltham, MA, USA), both with a TriVersa NanoMate (Advion, Ltd., Harlow, UK) source in LC chip coupling mode. In experiment 1, the MS was set on Top Speed for 3 s using the Orbitrap analyzer for MS and MS/MS scans with higher energy collision dissociation (HCD) fragmentation at a normalized collision energy of 28%. MS scans were measured at a resolution of 120,000 in the scan range of 375–1575 *m/z*. The MS ion count target was set to 1 × 10^6^ at a maximum injection time of 80 ms. The most intense peaks (charge state 2–6) were isolated for MS/MS scans by a quadrupole with an isolation window of 1.6 *m/z* and were measured with a resolution of 30,000. The automatic gain control target was set to 1 × 10^5^ with a maximum injection time of 100 ms. In experiment 2, mass spectrometer full scans were measured in the Orbitrap mass analyzer within the mass range of 350–1550 *m/z*, at 60,000 resolution using an automatic gain control target of 3 × 10^6^ and maximum fill time of 50 ms. An MS/MS isolation window for ions in the quadrupole was set to 1.4 *m/z*. MS/MS scans were acquired from the top ten ions of charge state 2–6 using the HCD mode at normalized collision-induced energy of 28%, with a resolution of 15,000, an automatic gain control target of 1 × 10^5^, and maximum injection time of 100 ms. In both experiments, the exclusion time to reject masses from repetitive MS/MS fragmentation was set to 30 s. The mass spectrometry proteomics data have been deposited to the ProteomeXchange Consortium (http://proteomecentral.proteomexchange.org, accessed on 11 January 2021) via the PRIDE partner repository [27] with the dataset identifier PXD018912.

### 2.8. Data Analysis

Proteome Discoverer (v2.1, Thermo Fischer Scientific) was used for protein identification and quantification. The acquired spectra were searched by Sequest HT against a *D. mccartyi* strain CBDB1 database containing 1454 non-redundant protein-coding sequence entries (downloaded October 2014 from UniProt) and a “common repository of adventitious proteins database” (cRAP) to exclude contaminants. Enzyme specificity was selected as trypsin with up to four missed cleavages allowed. Peptide ion tolerance was set to 10 ppm and MS/MS tolerance to 0.02 Da. Acetylation of lysines and protein N-termini and oxidation of methionines were selected as dynamic and carbamidomethylation of cysteines as static modification. A maximum of three equal and four dynamic modifications per peptide were allowed. Only peptides with a false discovery rate (FDR) < 0.01, calculated by Percolator, and a Sequest HT XCorr > 2.0 were considered as identified. Quantification of proteins was performed using the average of the top three peptide MS1 areas. Protein quantification was considered successful for proteins quantified in > 50% (i.e., two of three) of biological replicates; otherwise, they were classified as identified proteins. After log10 transformation, protein abundances were median-normalized and scaled, so that the global minimum is zero. MS1 areas of acetylated peptides which only differ in their modification status (oxidation) were summed and counted as one acetylation site. To correct acetylation differences for different protein amounts, estimated log10 acetylation abundance ratios were obtained by subtracting the median-normalized log10 MS1 peak area of the respective protein (measured in the total proteome) from the log10 MS1 peak area of the acetylated peptide (measured in the Kac-eluate).

Functional annotation was conducted using GhostKOALA [28]. KOALA (KEGG Orthology And Links Annotation) assigns K numbers, i.e., specific pathways, to proteins or genes, based on their BLAST search similarity to experimentally and functionally characterized genes. The significance of protein abundance fold changes and pathway coverage fold changes was calculated using two-tailed, homoscedastic Student’s *t*-tests. Raw *p*-values from protein comparisons were adjusted according to Benjamini–Hochberg. Figures were created using R software v.3.6.1 and the package ggplot2 [29,30].

## 3. Results and Discussion

### 3.1. Proteome and Acetylome in Different Growth Phases

*D. mccartyi* strain CBDB1 was grown in two-liquid phase cultures with 1,2,3-TCB as the electron acceptor. To obtain sufficient cell material for proteome and acetylome analysis (10^9^ cells) of the exponential and stationary growth phases, two individual culture setups were inoculated with 4 × 10^6^ cells mL^−1^. Growth was followed in several replicate cultures each (Figure S1a, curves I and II). The release of chloride through reductive dechlorination of 1,2,3-TCB (Figure S1b) is exemplarily shown for the cultures that were harvested in the exponential phase (Figure S1a, curve I). The proteome and acetylome were analyzed in both growth phases. The total number of identified and quantified proteins was 948 and 1015 in the exponential and stationary growth phase, respectively. Acetylated proteins were identified in both growth phases. The relative proportion of proteins with at least one acetylated lysine in one of three replicates increased from 5% in the exponential to 11% in the stationary phase (Table S2A).

**Figure 1:**
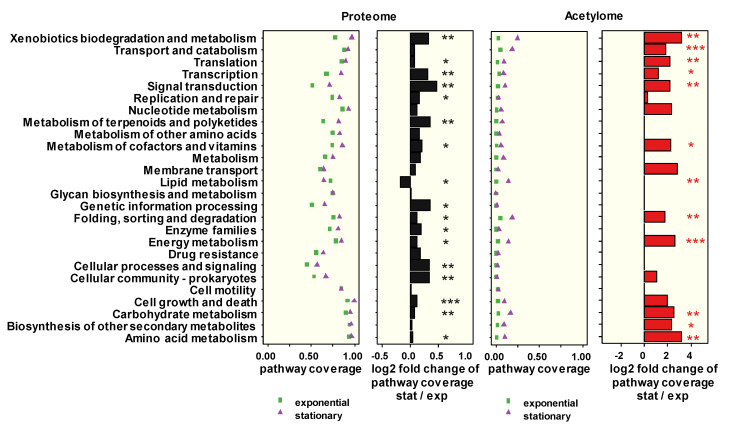
Global functional analysis of quantified proteins (left two columns) and acetylated proteins (right two columns). The displayed pathway coverage corresponds to the ratio of the number of quantifiable proteins or identified acetylated proteins, and the number of proteins encoded in the genome assigned to each pathway. The log2 fold changes were calculated from the pathway coverages of the two growth phases. Please note that the log2 fold changes of pathway coverages of the acetylome are zero if no acetylated proteins were detected in the exponential phase. Significance of differences between exponential and stationary phase is displayed as asterisks (* *p* < 0.05, ** *p* < 0.01, *** *p* < 0.001).

In total, 1042 different proteins were detected in at least one growth phase (Table S2A). They were subjected to a functional analysis using GhostKOALA [28]. Of these proteins, 64% were assigned to functional pathways based on their orthology to functionally characterized proteins (Table S2A). Among the remaining 36% of proteins not recognized by the bioinformatics tool were 150 uncharacterized proteins, as well as those predicted to be involved in organohalide respiration, such as reductive dehalogenases (see below). To analyze the importance of the identified pathways for a specific growth phase, the pathway coverages were compared (Figure 1).

A high pathway coverage (>0.8) was observed in both growth phases for the functions “transport and catabolism”, “translation”, “nucleotide metabolism”, “carbohydrate metabolism”, “biosynthesis of secondary metabolites”, and “cell growth and death”. The latter function also showed the most significant log2 fold change of pathway coverage between the stationary and the exponential growth phases, indicating the different physiological states of these cells. Noticeably, the log2 fold changes of the pathway coverages of stationary versus exponential phase cells were mostly positive, indicating a generally increased coverage of the functional pathways in stationary phase cells.

Although only a low amount of protein was available for the enrichment of lysine-acetylated peptides, altogether 192 unique lysine-acetylated peptides were detected in the two growth phases (Table S2B). Of these, 88 peptides were quantifiable, which represented 65 individual proteins with one to six quantifiable N_ε_-lysine-acetylated peptides. The majority of the quantifiable acetylated peptides (79) was found in the stationary phase, with only 19 in the exponential phase (Table S2B). This is in accord with reports for several other bacteria, e.g., *Escherichia coli* [21], *Mycobacterium tuberculosis* [31], *Clostridium acetobutylicum* [32], and *Borrelia burgdorferi* [33]. The pathway coverage of acetylated proteins was generally low (< 0.25, Figure 1), suggesting a post-translational regulation of the respective functional pathways only at specific positions. Among the pathway coverages with the highest log2 fold change in the stationary phase were the functional pathways “translation”, “transport and catabolism”, “folding, sorting and degradation”, “membrane transport, “carbohydrate metabolism”, “energy metabolism”, and “lipid metabolism” (Figure 1). These observations correspond well with the reported main functional targets in a range of other bacteria [34,35,36]. One remarkable difference between the exponential and stationary phase was the number of acetylated proteins with a function in “xenobiotics biodegradation and metabolism”, the coverage of which was much higher in the stationary phase (Figure 1). However, a detailed examination revealed that GhostKOALA also assigned the pyruvate-ferredoxin oxidoreductase and an IMP dehydrogenase family protein to this functional group (Table S2B). However, in strain CBDB1, these proteins play a role in carbohydrate and nucleotide metabolism, respectively, rather than in the biodegradation of xenobiotics (Table S2A). This shows the need to carefully consider individual protein acetylations in the context of their presumed functions in *D. mccartyi*. To identify the most strongly acetylated proteins, a log10 acetylation abundance ratio was estimated for all acetylated peptides (Table S2B). Proteins containing an acetylated peptide with an estimated acetylation abundance ratio of ≥−1 (the MS1 peak area of the acetylated peptide corresponded to at least 10% of the MS1 peak area of the total protein) are summarized in Table 1, while those with a lower acetylation abundance ratio but with multiple acetylated peptides are included in Table 2. The highest ratio with 0.84 (~7-fold amount of the acetylated peptide over the unmodified protein) was found for a K^114^-containing peptide of the acetyltransferase CbdbA937 (GNAT family) in the stationary phase (Table 1). In the exponential phase, the acetylation was not quantifiable, suggesting a role for this enzyme in the growth phase-dependent acetylation status of the overall proteome. Another predicted GNAT family acetyltransferase, CbdbA951 (with a lower estimated log10 abundance ratio of −1.02, Table 1), was acetylated at K^2^, but only in the exponential growth phase, suggesting a different function in the cell. The biochemically characterized acetyltransferase PatZ of *E. coli* is known to undergo autoacetylation by acetyl-CoA in a cooperative manner, which promotes its oligomerization to an octameric state, in which it is more stable and active than as a tetramer [37]. PatZ is responsible for the acetylation of the acetyl-CoA synthetase (Acs). By analogy, we hypothesize that CbdbA937 becomes Nε-lysine-acetylated when *D. mccartyi* transitions to the stationary phase, and this is potentially governed by the accumulation of acetyl-CoA in the cytoplasm due to the reduced biosynthetic activity of the cells. Accordingly, we could also observe multiple acetylations of *D. mccartyi* Acs CbdbA1126 only in the stationary phase (Table 2) and will discuss below the possible metabolic consequences in the context of the regulation of central carbon metabolism.

The other strongly acetylated proteins were assigned to different functional pathways (Table 1), suggesting that their post-translational modification impacts central metabolic functions such as the biosynthesis of terpenoids (2-C-methyl-D-erythritol 4-phosphate cytidylyl-transferase), metabolism of cofactors and vitamins (GTP cyclohydrolase, FolE), transcription (transcription elongation factor GreA), replication (DNA gyrase subunit B), carbohydrate metabolism (pyruvate-ferredoxin oxidoreductase, gamma-subunit), and membrane transport of proteins (twin-arginine translocation protein TatA). All proteins are either only, or more strongly, acetylated in the stationary phase.

Fourteen further proteins showed multiple acetylations (Table 2). They contained up to 11 acetylation sites, of which up to 6 could be quantified. With the exception of CbdbA309, all of the gene products belong to the most highly abundant proteins in the proteomes (average log10 unmodified protein abundance 3.3 ± 0.5 vs. 3.0 ± 0.6 in all proteins). These protein acetylations show slight variation depending on the growth phase, suggesting that they may be an important mechanism for modulating protein activity. The respective proteins are associated with “folding, sorting and degradation”, “translation”, and “carbohydrate metabolism”. Remarkably, three proteins belong to the latter function: Acs, the myo-inositol-1-phosphate synthase, and glyceraldehyde-3-phosphate dehydrogenase.

**Figure 2:**
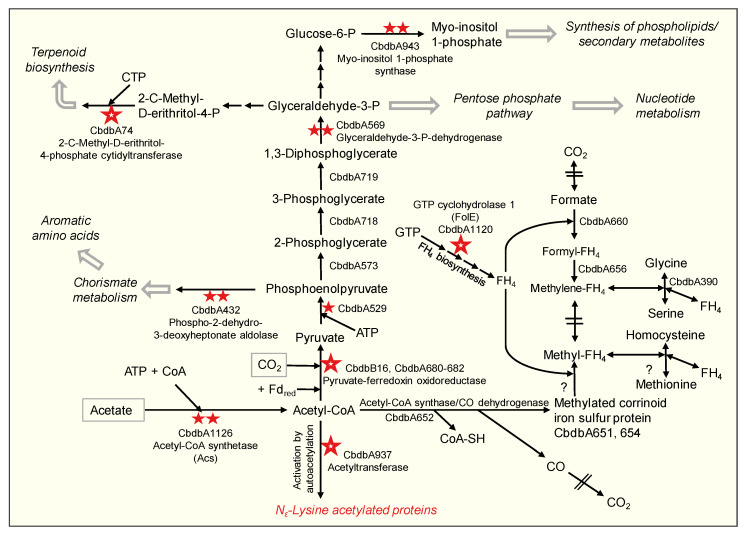
Carbon metabolism of *Dehalococcoides mccartyi* strain CBDB1 demonstrating the main routes of acetate utilization via acetyl-CoA, gluconeogenesis, and acetyl-CoA synthase/CO dehydrogenase (black arrows) and the diverging biosynthetic pathways (open arrows). Nε-lysine-acetylated proteins are indicated by red asterisks: A high acetylation abundance ratio is indicated by a large asterisk (see Table 1), while multiple acetylation sites are denoted by two smaller asterisks (Table 2) and less abundant acetylation by a single, small asterisk (Table S2). The incomplete Wood–Ljungdahl pathway displayed on the right is a source of C1 carriers, needed e.g., for the synthesis of methionine and serine, and might be strongly influenced by the availability of tetrahydrofolate (FH_4_). The locus tags of the enzymes annotated in the genome of strain CBDB1 are displayed. The *?* signifies that enzymatic activity was demonstrated; however, to date, the corresponding enzyme has not been annotated [38].

The pronounced acetylation of enzymes of the central carbon metabolism has already been recognized in the first analyses of prokaryotic Nε-lysine acetylation (e.g., [21,35]). Figure 2 schematically shows the carbon metabolism of *D. mccartyi* with the positions of the respective acetylated proteins. The Acs is central to the metabolism of *D. mccartyi*. It converts the carbon source acetate in a two-step reaction into acetyl-CoA, which in turn is converted into pyruvate, the starting point for gluconeogenesis (Figure 2). Acetyl-CoA is also the substrate for the acetyl-CoA synthase/CO dehydrogenase complex and provides the C1 group for the formation of methyltetrahydrofolate in the incomplete Wood–Ljungdahl pathway. The growth-inhibiting carbon monoxide can also be released as a side product [38] (Figure 2). It is known that in *Salmonella enterica*, the activity of Acs is regulated by the reversible acetylation and deacetylation of its lysine residue in the active center [39], which is responsible for the catalysis of the first half-reaction that consumes ATP [37]. In *D. mccartyi*, Acs is acetylated at multiple positions (Table 2). It is therefore conceivable that a downregulation of the Acs activity by acetylation helps with the transition to the stationary phase and at the same time, protects the bacterium by reducing or preventing the release of CO by the acetyl-CoA synthase/CO dehydrogenase complex [38].

It is known that glycolytic enzymes contain a particularly large number of evolutionarily conserved acetylated lysine residues in their active centers [36]. *D. mccartyi* cannot utilize glucose or other sugars and, therefore, uses the glycolytic cascade in the reverse direction for gluconeogenesis. Interestingly, only a few of the glycolytic enzymes were acetylated in strain CBDB1, e.g., CbdbA529, the phosphoenolpyruvate synthase (Figure 2, Table S2B) but not at an evolutionarily conserved lysine. However, the pyruvate-ferredoxin oxidoreductase (particularly the gamma subunit CbdbB16) was strongly acetylated (Figure 2, Table 1), suggesting the downregulation of an early step in gluconeogenesis during the stationary phase. Interestingly, glyceraldehyde-3-phosphate dehydrogenase (GAPDH) was also acetylated (Figure 2, Table 2). Wang et al. [35] demonstrated the inhibition of its gluconeogenic activity by acetylation in *S. enterica*. By analogy, acetylation of the GAPDH in *D. mccartyi* might have implications for subsequent glucose 6-phosphate synthesis (Figure 2). The latter can also be converted by myo-inositol 1-phosphate synthase (CbdbA943), which also had two acetylated lysine residues in the stationary phase (Table 2). These acetylations, therefore, possibly influence phospholipid biosynthesis.

In contrast to the facultative organohalide-respiring *Sulfurospirillum multivorans* [24], in *D. mccartyi* the transcriptional regulators putatively involved in OHR were not acetylated. However, the identified strong acetylation (Table 1) of the transcription regulator GreA [40] in the stationary phase might result in a global downregulation of transcription, probably also contributing to the survival of the obligate OHR bacterium under non-growth conditions.

One significant finding was the high estimated acetylation abundance ratio of TatA (CbdbA1694, Table 1) exclusively in the stationary phase. It is a component of the twin-arginine translocation machinery, which transports folded proteins across the cytoplasmic membrane. The annotated Tat operon of strain CBDB1 encodes a second copy of TatA, CbdbA1695, which was not acetylated and might possess a different substrate specificity than its counterpart. To our knowledge, acetylation of Tat components has so far not been reported in the literature. The strong acetylation of TatA in the stationary phase would be consistent with its role in the energy metabolism of *D. mccartyi*. This depends on a functional OHR complex, the main components of which, RdhA, HupLS and OmeA, are (co)translocated to the cytoplasmic membrane and the periplasm by the Tat translocon [41,42]. TatA is the component that is thought to permeabilize the membrane for the transport of the folded protein substrates, which are bound by their signal peptide to the TatBC translocon core components [43,44]. TatA is anchored with its N-terminus in the cytoplasmic membrane, and an adjacent amphipathic helix that is followed by a largely unstructured C-terminal domain is located on the cytoplasmic side of the membrane [45]. Upon binding of the protein substrate to TatA, a conformational change leads to membrane weakening, which is thought to be a prerequisite for the transport of the folded protein [46]. *D. mccartyi* might have adopted this lysine modification to maintain membrane integrity in the stationary phase by reducing Tat-dependent transport. For example, one might speculate that the strong C-terminal (K^66^) acetylation of TatA interferes with its substrate binding or conformational change, which would normally induce membrane destabilization upon substrate association. In both cases, the proton gradient-consuming, and therefore, energetically demanding, transport of folded proteins would be inhibited.

In addition to TatA, the main RdhAs CbdbA80 and CbrA were also found to be acetylated. Three different acetylation sites were identified in RdhA CbdbA80 (Table S2B). Only one, K^365^, was quantifiable in the stationary phase. In the case of CbrA, one acetylated peptide was identified in the stationary phase (Table S2B). Although the mature OHR complex is located in the membrane and is likely not to be directly targeted, the observed acetylation might have occurred during folding and prior to transport of the dehalogenases across the membrane. Interestingly, K^365^ of RdhA CbdbA80 is located near one of the two Fe–S cluster binding regions (Figure S2), suggesting a negative impact on RdhA maturation and activity. Acetylation might suggest the cytoplasmic storage of excess RdhAs in stationary phase cells, which could then, through deacetylation and transport, enable rapid onset of OHR.

### 3.2. The Organohalide Respiration Complex

**Figure 3:**
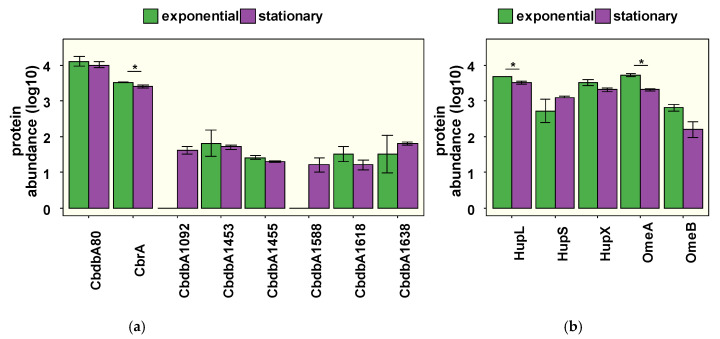
Median-normalized protein abundances of RdhAs (**a**) and other components of the OHR complex (**b**) in the exponential and stationary growth phases of the first experiment. The small, extremely hydrophobic RdhB proteins were not detected in any proteome analysis. Mean values and standard deviations of triplicate cultures are shown; id, identified in one replicate; *, Benjamini–Hochberg adjusted *p*-values <0.05.

The main components of the OHR complex—the RdhAs, the predicted molybdoenzyme OmeA, the subunits of the uptake NiFe hydrogenase, HupL and HupS, as well as the iron–sulfur cluster binding protein HupX—all belong to the most abundant proteins of the proteome in both growth phases (Table S2A). Eight different RdhAs were detected in the proteomes during growth (Figure 3a). The RdhAs CbrA and CbdbA80 were the most abundant. They were among the 2% most abundant proteins of the entire proteome with median-normalized relative log10 abundances of 3.6 to 4.2. Although present at relative abundances that were two orders of magnitude lower, the RdhAs CbdbA1455, CbdbA1453, CbdbA1618, and CbdbA1638 were also present in exponential and stationary phase cells, whereas the two other low-abundance RdhAs, CbdbA1092 and CbdbA1588, were detected only in the stationary phase. To verify the stable abundance of the main components of the OHR complex during growth, a second set of cultures was grown (Figure S3). The data confirmed the stable presence of the most abundant RdhAs CbdbA80 and CbrA in both growth phases (Figure S4a). The less abundant RdhAs (log10 abundance ≤2) showed some variability; however, the presence of CbdbA1453, CbdbA1455, CbdbA1618, and C1638 in at least one growth phase was confirmed. Instead of the two minor RdhAs identified in experiment 1, CbdbA1092 and CbdbA1588, a different RdhA (CbdbA187) was identified in the exponential growth phase. Such variations in the detection of low-abundance RdhAs have already been observed recently [2]. The putative membrane anchor proteins RdhB were not detected in either experiment, and the membrane-integral protein OmeB was less abundant, probably because their solubilization from the membrane requires a detergent treatment [3], which was omitted in this study, and is a general challenge in proteome studies [47]. However, the other components of the OHR complex—HupS, HupL, HupX, and OmeA—were also abundant in the second experiment and throughout growth (Figure 3b and Figure S4b). To test the stability of this complex over longer periods, we also analyzed the proteome of the four-week-old stationary phase cultures used as inoculum and compared this with a freshly inoculated control culture incubated for five days without 1,2,3-TCB. Interestingly, the abundances of CbdbA80, CbrA, HupL, and OmeA were unchanged in both the inoculum and the control culture without 1,2,3-TCB (Figure S4, Table S3). This suggests an astonishingly high stability of the OHR complex components in stationary phase cells and during culture manipulations. Such stability can be seen as “life insurance” for this highly specialized bacterium, since the OHR complex is essential for the long-term generation and maintenance of a proton gradient in stationary phase cells. Hence, growth can be initiated immediately after the emergence of suitable conditions such as access to halogenated compounds. The localization of the OHR complex on the outer face of the cytoplasmic membrane might contribute to its long-term stability; however, mechanisms maintaining its long-term retention of integrity and function are not yet understood.

**Figure 4:**
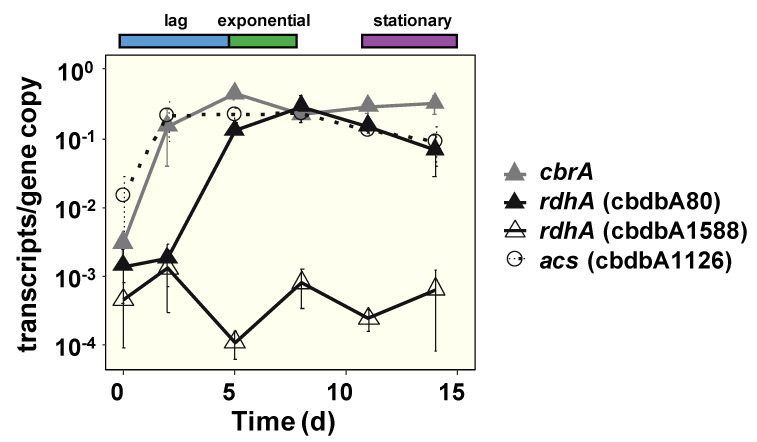
Transcription of *rdhA* genes *cbrA*, cbdbA80, and cbdbA1588, as well as of the gene cbdbA1126 encoding the acetyl-CoA synthetase of *D. mccartyi* strain CBDB1 was analyzed in the cultures shown in Figure S3, curve III. The respective ranges of the lag, exponential, and stationary phases are indicated. Mean values and standard deviations of three replicates are shown.

It was shown previously that the transcription of the *rdhA* genes was induced in the presence of the cognate halogenated electron acceptor [18,48]. To study the possible contribution of transcription to the observed high abundance of RdhAs throughout growth, the mRNA of selected *rdhA* genes was quantified in two-liquid phase cultures in the second experiment, in which cells were again incubated with 1,2,3-TCB (Figure S3, curve III). The transcript level of the *rdhA* genes *cbrA* and cbdbA80 increased 100-fold within the first five days before growth was initiated and expression levels remained relatively unchanged thereafter (Figure 4). In contrast, transcription of the *rdhA* gene cbdbA1588 was not induced, correlating with the absence of the corresponding protein in this experiment (Figure S4a). For comparison, the expression of the central house-keeping *acs* gene, cbdbA1126, increased only 10-fold in the first two days of cultivation and remained stable and unchanged until harvest. This emphasizes the strong transcriptional upregulation of the two *rdhA* genes, which encode the most abundant RdhAs in the proteome. They obtained their highest transcript level when growth was initiated, so that *de novo* protein synthesis in the exponential phase is ensured and can compensate for the dilution of the RdhAs during cell proliferation.

## 4. Conclusions

The findings of this study reveal changes in both the proteome and acetylome of *D. mccartyi* strain CBDB1 during the transition between exponential and stationary phase cells. Because *D. mccartyi* has evolved to exploit limiting concentrations of organohalides as electron acceptors, such a study provides initial insights into how a bacterium copes with survival at the energy limit. The proteome and acetylome showed remarkable changes during growth, which likely confers upon the *D. mccartyi* strain CBDB1 the metabolic flexibility necessary to adapt to such restrictive environmental conditions. The increased number of acetylated proteins at key nodes of primary metabolism, as well as of membrane transport (e.g., TatA), which is necessary for establishment of the abundant OHR complex, suggests that protein acetylation is a means of rapidly modulating protein conformation and activity. The unexpectedly high abundance, yet apparent longevity of the OHR complex, suggests an overall energy- and resource-saving downregulation of metabolism in the natural environment when essential factors for organohalide respiration become limiting. Although preliminary, our study provides us with a basis to study the survival of this highly specialized bacterium when the prerequisites for growth are absent in the short- or long-term. The development of such a model system will aid our general understanding of dormancy and how microorganisms have evolved to survive even with limited possibilities for energy conservation.

## Figures and Tables

**Table 1:** Highly acetylated proteins (i.e., estimated log10 acetylation abundance ratio ≥ −1.1 ^a^).

Protein Description	Locus Tag	Pathway	Acetylated Lysine	Estimated log10 Acetylation Abundance Ratio	
				Exponential Phase	Stationary Phase
Acetyltransferase, GNAT family	cbdbA937	No pathway assigned	K^114^	*id.*	0.84
Acetyltransferase, GNAT family	cbdbA951	No pathway assigned	K^2^	−1.02	*n.d.*
2-C-methyl-D-erythritol 4-phosphate cytidylyl-transferase	cbdbA74	Metabolism of terpenoids and polyketides	K^138^	*n.d.*	−0.07
GTP cyclohydrolase 1	cbdbA1120	Metabolism of cofactors and vitamins	K^104^	*id.*	−0.26
Twin-arginine translocation protein TatA	cbdbA1694	Folding, sorting, and degradation; membrane transport	K^66^	*n.d.*	−0.66
Transcription elongation factor GreA	cbdbA743	Transcription	K^17^	−1.56	−0.76
Pyruvate-ferredoxin oxidoreductase, gamma-subunit	cbdbB16	Carbohydrate metabolism; energy metabolism; xenobiotics biodegradation and metabolism	K^166^	*id.*	−0.95

^a^ Refers to an acetylation ratio of ≥ 10%, calculated from the MS1 peak areas of the respective acetylated peptide and the non-acetylated protein; *n.d.* not detected, *id.* Acetylated peptide identified in ≥1 replicates or quantified in 1 replicate.

**Table 2:** Proteins with multiple quantifiable acetylation sites.

Protein Description	Locus Tag	Pathway	No. of Detected Acetylated Peptides	Acetylated Lysines within Quantifiable Peptides	Estimated log10 Acetylation Abundance Ratio	
					Exponential Phase	Stationary Phase
60 kDa chaperonin GroEL	CbdbA1393	Folding, sorting, and degradation; translation; transport and catabolism	11	K^326^ or K^327^ K^121^ K^10^ K^116^ K^465^ K^362^	*n.d.* *n.d.* *id.* −3.13 *n.d.* −2.63	−2.91 −2.45 −2.35 −2.49 −2.89 id.
10 kDa chaperonin GroES	CbdbA1392	Folding, sorting, and degradation; translation	3	K^66^ K^52^ or K^55^ K^19^	*n.d.* *n.d.* *n.d.*	−2.29 −2.53 −2.78
Elongation factor TU	CbdbA960	Translation; transport and catabolism	10	K^57^ K^377^ K^219^	−3.46 id. id.	−2.84 −2.67 −2.61
Uncharacterized protein	CbdbA727	No pathway assigned	5	K^100^ K^97^ or K^100^	*n.* *d.* *n.d.*	−2.48 −2.50
Conserved domain protein	CbdbA1024	No pathway assigned	5	K^184^ K^132^ K^18^ K^425^	−2.26 −2.51 *n.d.* −2.72	−1.59 *id.* −2.04 *id.*
Myo-inositol-1-phosphate synthase family protein	CbdbA943	Biosynthesis of other secondary metabolites; carbohydrate metabolism	3	K^110^ K^240^	*n.d.* *n.d.*	−1.61 −2.44
Phospho-2-dehydro-3-deoxyheptonate aldolase	CbdbA432	Amino acid metabolism	4	K^98^ K^26^	*n.d.* *n.d.*	−2.17 −2.67
Acetyl-CoA synthetase	CbdbA1126	Carbohydrate metabolism; energy metabolism; lipid metabolism	3	K^573^ K^518^ K^7^	*n.d.* *n.d.* *n.d.*	−1.95 −2.46 −1.77
Conserved domain protein	CbdbA688	No pathway assigned	4	K^82^ K^65^	*id.* −*2.51*	−1.99 *id.*
GTPase domain protein	CbdbA568	No pathway assigned	3	K^367^ K^256^	−2.64 *n.d.*	*id.* −1.96
Glyceraldehyde-3-phosphate dehydrogenase, type I	CbdbA569	Carbohydrate metabolism; energy metabolism; signal transduction; transport and catabolism	3	K^334^ or K^335^ K^195^	*n.d.* *n.d.*	−1.46 −1.82
30S ribosomal protein S18	CbdbA1018	Translation	2	K^114^ K^131^ or K^135^	*id.* *n.d.*	−1.67 −2.17
Inorganic pyrophosphatase	CbdbA309	Energy metabolism	2	K^30^ K^205^, K^208^ or K^209^	*n.d.* *n.d.*	−1.53 −1.81
NifU protein, homolog	CbdbA1730	No pathway assigned	3	K^1^ or K^4^ N-Terminus	−2.58 −1.46	*n.d.* −1.66

*n.d.* not detected, *id.* Acetylated peptide identified in ≥1 replicates or quantified in 1 replicate.

## Data Availability

The mass spectrometry proteomics data have been deposited to the ProteomeXchange Consortium (http://proteomecentral.proteomexchange.org, accessed on 15 February 2021) via the PRIDE partner re-pository [27] with the dataset identifier PXD018912.

## Supplementary Materials

The following are available online at https://www.mdpi.com/2076-2607/9/2/365/s1: Table S1: Primer and plasmids used for the quantification of gene transcription. Figure S1: Growth curves of the two culture set-ups used to harvest cells from the exponential and stationary phase and release of chloride during reductive dechlorination of 1,2,3-TCB. Table S2: Basic proteomic and acetylomic datasets from all samples of experiment 1. Figure S2: The location of the acetylation at K365 of CbdbA80. Figure S3: Growth curves for cultures devised for the second proteome experiment and the transcription analysis. Figure S4: Abundances of detected RdhAs and components of the OHR complex in the second proteome experiment. Table S3: Basic proteomic datasets from all samples of experiment 2.
