# Supplementary material for: Changes of the Proteome and Acetylome during Transition into the Stationary Phase in the Organohalide-Respiring Dehalococcoides mccartyi Strain CBDB1
Source: Microorganisms. 2021 Feb 12;9(2):365. doi: 10.3390/microorganisms9020365 (PMC7918482; doi:10.3390/microorganisms9020365)
Supplement: Supplementary file 1 [file microorganisms-09-00365-s001.zip › Supplementary_Figures_S1-S4,Table_S1.pdf]

Changes of the Proteome and Acetylome during Transition into the Stationary Phase in the Organohalide-Respiring *Dehalococcoides mccartyi* Strain CBDB1

Franziska Greiner–Haas[1], Martin von Bergen[2], Gary Sawers[1], Ute Lechner[1]*, Dominique Türkowsky[2]*

[1]Institute of Biology/Microbiology, Martin-Luther University Halle-Wittenberg, Halle, Germany;
[2]Department of Molecular Systems Biology, Helmholtz Centre for Environmental Research – UFZ, Leipzig, Germany

*Corresponding authors.

Supplementary data

Table S1. Primer and plasmids used for the quantification of gene transcription.

Primer	Sequence 5' → 3'	Target gene (locus tag)	Size of the PCR product (bp)
Luci_f[a]	TTCCAGGGATACGACAAGG	*Photinus pyralis* luciferase cDNA	200
Luci_r[a]	CGGACATAATCATAGGTCCT		
Ac-CoA _f	GTAGATGAGGCAGTCAAATC	*acs* (cbdbA1126)	122
Ac-CoA_r	GCATCTTTAAGCAAATCATG		
cbdbA80_f	ATGCCTGTCCCGGTAACG	*rdhA* (cbdbA80)	190
cbdbA80_r	CAAGTACCCTGACAAACTCC		
cbdbA84_f[a]	CTTATATCCTCAAAGCCTGA	*cbrA* (cbdbA84)	201
cbdbA84_r[a]	TGTTGTTGGCAACTGCTTC		
cbdbA1588 _f[a]	CTGAAAGGAATAGGTCTGG	*rdhA*(cbdbA1588)	194
cbdbA1588.01_r	CAAGTGTAAGGGCGTCTTTG		

Plasmids	Insert description [position in the genome of strain CBDB1]
pLUCI[a, b]	Partial sequence of *Photinus pyralis* luciferase cDNA
pJET_A80	Partial sequence of *rdhA* (cbdbA80) [62238-62853]
pCBDBA84[a, b]	Partial sequence of *cbrA* (cbdbA84) [67640-67841]
pCBDBA1588	Partial sequence of *rdhA* (cbdbA1588) [1275489-1275705]
pJET_A1126	Partial sequence of *acs* (cbdbA1126) [915830-916415]

[a]see Wagner et al. 2009 [1]. Plasmids were derived by standard cloning procedures from the vectors pJET1.2 (Thermo Fisher Scientific, Schwerte, Germany) and [b]pGEM T-Easy (Promega, Walldorf, Germany).

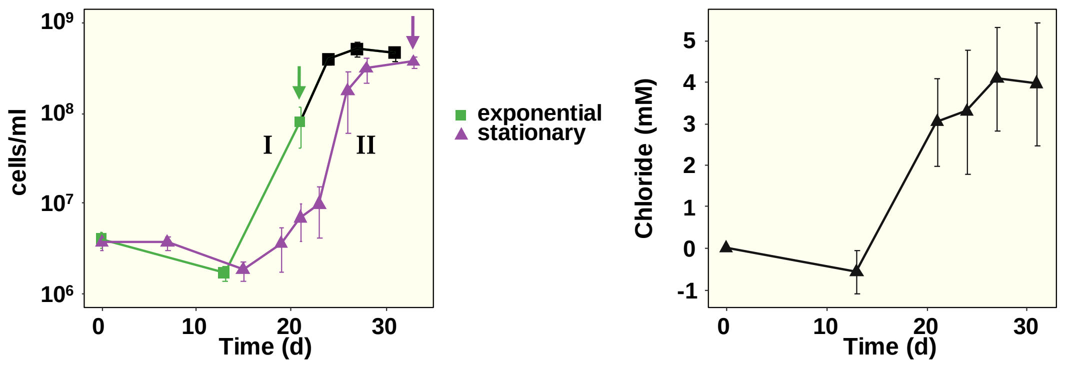

(a) (b)

Figure S1. Growth curves (a) of the two culture set-ups used to harvest cells from the exponential (I) and stationary phase (II). The arrows indicate the sampling points for proteome and acetylome analysis. Shown are the mean values and standard deviations of six and three replicates for culture set-ups I and II, respectively. After harvesting three cultures for the analysis of the exponential growth phase (curve I, green line), growth was further monitored in the three remaining culture replicates (curve I, black line). (b) Release of chloride during reductive dechlorination of 1,2,3-TCB (initial nominal concentration 10 mM) in culture set-up I. The chloride concentration was determined using a chlor-o-counter instrument (FLOHR Instruments, Nieuwegein, The Netherlands). For clarity, the measured values were normalized by subtracting the chloride concentration of the medium at day zero.

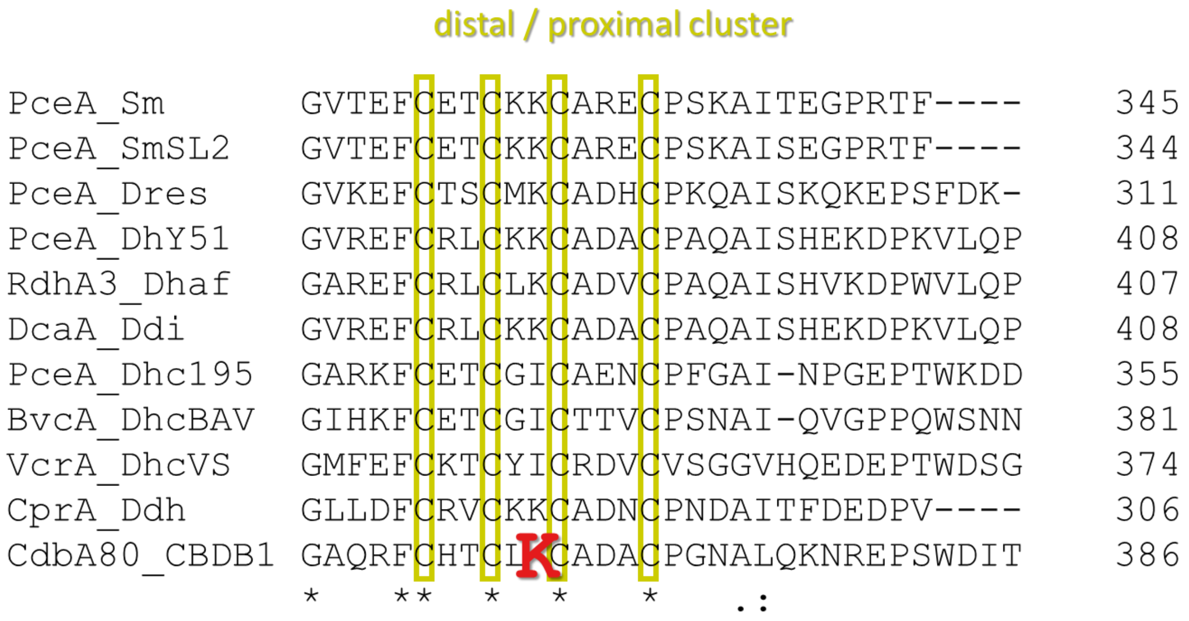

Figure S2. The acetylation at K^{365} (red) of CbdbA80 in the stationary phase is located in a conserved Fe/S-cluster binding region. The lysine was localized by alignment with sequences of known reductive dehalogenases, among which is one with a described structure according to Bommer et al. 2014 [2].

Selected reductive dehalogenases from the following organohalide-respiring bacteria were aligned with Clustal Omega: PceA_Sm - *Sulfurospirillum multivorans* PCE/TCE RDase (AF022812), PceA_SmSL2 *Sulfurospirillum* sp. SL2 PCE-only RDase (AGW23615), PceA_Dres *Dehalobacter restrictus* DSM 9455 PCE/TCE RDase (AHF10423), PceA_DhY51 *Desulfitobacterium hafniense* Y51 PCE/TCE RDase (YP519072), RdhA3_Dhaf *D. hafniense* DCB-2 dichlorophenol (DCP)/PCE RDase (YP002457196), DcaA_Ddi *Desulfitobacterium dichloroeliminans* LMG P-21439 dichloroethane (DCA) RDase (CAJ75430), PceA_Dhc195 *Dehalococcoides mccartyi* 195 PCE/TCE RDase (DET0318), BvcA_DhcBAV *D. mccartyi* BAV1 TCE/DCE/DCA RDase (AAT48558), VcrA_DhcVS *D. mccartyi* VS vinyl chloride reductase (AAQ94119), CprA_Ddh *Desulfitobacterium* sp. PCE-1 o-chlorophenol reductive RDase (AF259790), CdbA80_CBDB1 *D. mccartyi* CBDB1 RdhA (CbdbA80).

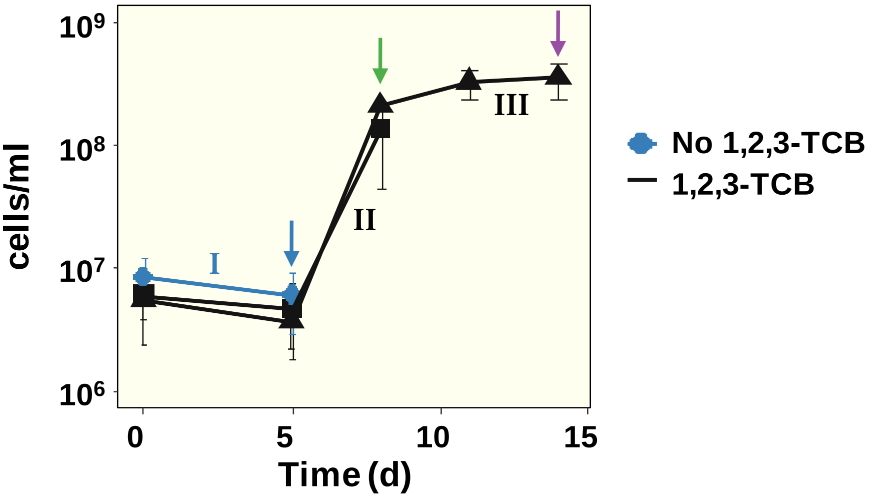

Figure S3. Growth curves for cultures devised for the second proteome experiment (Fig. 3) and the transcription analysis (Fig 4). The two-liquid phase cultures were incubated with 10 mM (nominal concentration) 1,2,3-TCB as the electron acceptor (II, III) or without the acceptor (I). Cultures I (12 replicates), II (6 replicates) and III (3 replicates) were harvested for proteome analyses. The sampling points are indicated by arrows (blue: without electron acceptor, green and purple: exponential and stationary growth phase, respectively). The cultures shown in curve III were additionally used for transcription analyzes. Mean values and standard deviations of three replicates are shown.

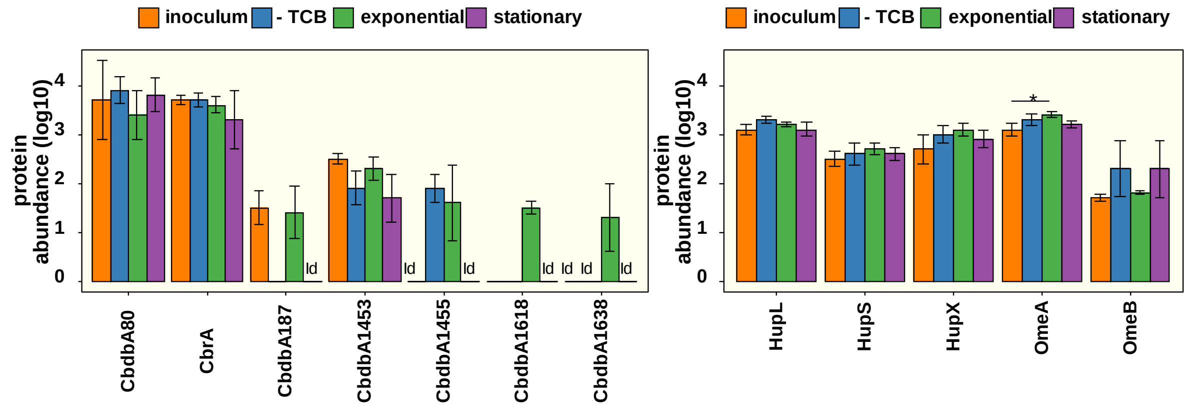

(a) (b)

Figure S4. Median-normalized abundances of detected RdhAs (a) and components of the OHR complex (b) in the second proteome experiment. The inoculum (cells of a four week-old stationary phase pre-culture) and a culture incubated for five days in the absence of 1,2,3-TCB (- TCB)were also analyzed. RdhB proteins were not detected in any proteome. The data confirmed the stable presence of the most abundant RdhAs CbdbA80 and CbrA in the exponential and stationary phases. They were similar abundant in the inoculum and in the absence of 1,2,3-TCB. The other main components of the OHR complex showed also only small variations of their abundance under the tested conditions. Mean value and SD of triplicate cultures; id: identified in at least one replicate (see also Table S3). *: Benjamini-Hochberg adjusted p-values <0.05.

References:

[1] A. Wagner, L. Adrian, S. Kleinsteuber, J.R. Andreesen, U.Lechner, Transcription analysis of genes encoding homologues of reductive dehalogenases in “*Dehalococcoides*” sp. strain CBDB1 by using terminal restriction fragment length polymorphism and quantitative PCR. Appl Environ Microbiol, 2009; 75: 1876-1884.

[2] M. Bommer, C. Kunze, J. Fesseler, T. Schubert, G. Diekert, H. Dobbek, Structural basis for organohalide respiration, Science 346 (6208) (2014) 455-458.
